# Extra-abdominal trocar and instrument detection for enhanced surgical workflow understanding

**DOI:** 10.1007/s11548-024-03220-0

**Published:** 2024-07-15

**Authors:** Franziska Jurosch, Lars Wagner, Alissa Jell, Esra Islertas, Dirk Wilhelm, Maximilian Berlet

**Affiliations:** 1 Research Group MITI, Klinikum rechts der Isar, TUM School of Medicine and Health, Technical University of Munich, Munich, Germany; 2 Department of Surgery, Klinikum rechts der Isar, TUM School of Medicine and Health, Technical University of Munich, Munich, Germany

**Keywords:** Trocar detection, Surgical workflow analysis, Surgical context awareness, Deep learning

## Abstract

**Purpose:**

Video-based intra-abdominal instrument tracking for laparoscopic surgeries is a common research area. However, the tracking can only be done with instruments that are actually visible in the laparoscopic image. By using extra-abdominal cameras to detect trocars and classify their occupancy state, additional information about the instrument location, whether an instrument is still in the abdomen or not, can be obtained. This can enhance laparoscopic workflow understanding and enrich already existing intra-abdominal solutions.

**Methods:**

A data set of four laparoscopic surgeries recorded with two time-synchronized extra-abdominal 2D cameras was generated. The preprocessed and annotated data were used to train a deep learning-based network architecture consisting of a trocar detection, a centroid tracker and a temporal model to provide the occupancy state of all trocars during the surgery.

**Results:**

The trocar detection model achieves an F1 score of $95.06\pm 0.88\%$. The prediction of the occupancy state yields an F1 score of $89.29\pm 5.29\%$, providing a first step towards enhanced surgical workflow understanding.

**Conclusion:**

The current method shows promising results for the extra-abdominal tracking of trocars and their occupancy state. Future advancements include the enlargement of the data set and incorporation of intra-abdominal imaging to facilitate accurate assignment of instruments to trocars.

## Introduction

Context awareness and context-based behavior of assistive systems are a key factor for generating added value and ensuring acceptance in the surgical field [1]. The generation of contextual information through surgical workflow analysis for laparoscopic interventions, e.g., by intra-operative tracking of surgical instruments, is a prominent research area [2, 3]. In comparison with electromagnetic or optical markers, video-based tracking solutions do not have a necessity for additional hardware on the instrument, characterized by elevated costs, space requirements, as well as demand for technical expertise [4]. Video-based tracking has been enabled primarily by recent advances in the field of artificial intelligence (AI), in particular by the further development of deep learning (DL) techniques such as convolutional neural networks (CNN) [5]. Hence, deep learning-based approaches for laparoscopic instrument detection in intra-abdominal video are very common [5]. However, a disadvantage of only relying on intra-abdominal videos is that instruments can only be detected when they are actually visible in the laparoscopic image. During surgery, laparoscopic instruments might not be visible but are still located in the abdominal cavity. Furthermore, when instruments are outside the abdomen, it is not possible to determine their position, whether at the operating table with the surgeon or on the instrument table with the operating room (OR) assistant. To enhance the situational awareness of assistive systems, we aim to develop a context-aware system that tracks which laparoscopic instrument is currently inserted in its corresponding trocar. Therefore, in this paper, we propose, to the best of our knowledge, the first video-based extra-abdominal trocar and instrument detection algorithm to determine whether an instrument is still in the abdomen. In a first step, we employ extra-abdominal cameras to detect and track trocars and assign their functionality to determine their occupancy state. In future steps, we aim to identify which instrument exactly is currently inserted.

## Related work

### Surgical workflow analysis and instrument detection

During the last 10 years, the interest in surgical workflow analysis has steadily increased, particularly since the availability of public data sets from intra-abdominal laparoscopic video sources such as Cholec80 or M2CAI16 [6]. The review of Demir et al. [2] provides an overview of different algorithms that have been tested for surgical workflow analysis, while the review of Wang et al. [5] focuses on algorithms for laparoscopic instrument detection and tracking. Boonkong et al. [7] showed good intra-abdominal instrument detection results using the one-stage detector YOLOv4. The approach by Alshirbaji et al. [8] included long short-term memory (LSTM) models using the Cholec80 dataset.

### Extra-abdominal tracking

Extra-abdominal instrument and trocar detection for laparoscopic interventions has not yet been investigated as intensively. Rosa et al. [9] used an external camera setup to infer the pivot point of laparoscopic instruments by recognizing an instrument shaft using a line detector approach. However, the approach was not tested in a surgical environment. Other methods have been presented by Birch et al. [10] and Dehghani et al. [11]. In order to autonomously approach trocars in robotically assisted vitreoretinal surgery, Birch et al. used ArUco markers placed next to the trocars and Dehghani et al. trained a U-Net-style network to recognize the significantly smaller eye trocars by a camera on the robotic setup. These approaches were also only tested in laboratory environments. Shimizu et al. [12] proposed a video-based extra-abdominal deep learning approach to detect instruments (scissors and needle holder) and surgeon’s hands in open plastic surgeries, using a data set of seven extra-abdominal videos recorded with a head-fixed camera during real surgeries. A two-stage Faster Region-based CNN model was used to detect the surgeon’s hands and instruments, and an LSTM model was used for tool labeling.

In contrast to most of the approaches presented above, this paper uses real surgical data and focuses on the extra-abdominal trocar detection in laparoscopic surgeries. Furthermore, we used two cameras to create the data set allowing for a multiview approach. Instead of a two-stage Faster Region-based CNN model for hand and instrument detection used by Shimizu et at., this architecture uses YOLOv8 for the trocar detection. For tracking, we use prior knowledge by limiting the number of trocars to be tracked to a maximum of four. This improves robustness against fast camera movements and restricts the switching of unique identifiers (ID).

## Methods

### Task formulation

**Fig. 1:**
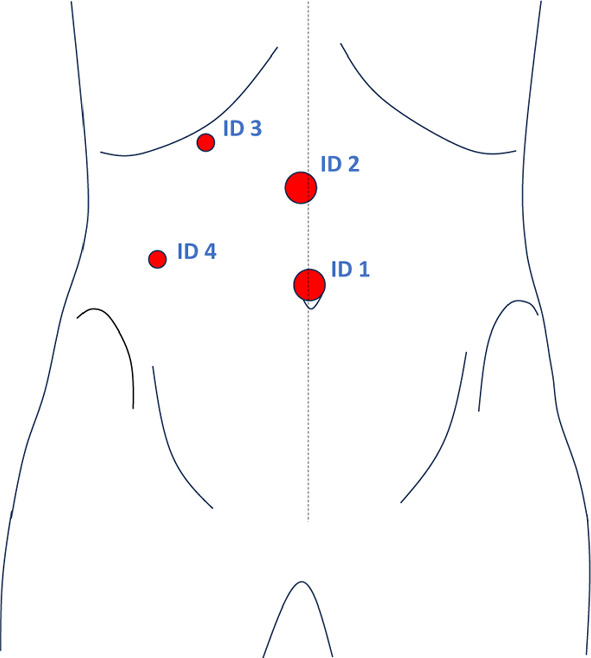
Trocar placement during laparoscopic cholecystectomies. The common positions of the trocars are shown in red. We assign each trocar a unique identifier. The size of the markers correlates with the diameter of the trocars. Trocars with ID1 and ID2 are usually 10 mm trocars, while trocars with ID3 and ID4 are 5 mm trocars

Figure 1 shows the trocar placement for a laparoscopic cholecystectomy. The camera trocar is usually placed in the median umbilical region. A trocar positioned in the lower right abdomen, along with another trocar placed paramedian on the right side in the epigastrium, are strategically used as the surgeon’s working trocars, respectively, to facilitate triangulation during the procedure. A palpation probe is introduced through an additional trocar positioned along the right subcostal medioclavicular line. This placement allows for the lever effect on the patient’s thorax, effectively keeping the liver lobe out of the field of view.

In this paper, we would like to detect these trocars in a first step by means of a camera placed in the lamp and allocate their positions. In addition, we would like to make a statement as to whether they are currently occupied with an instrument enhancing context awareness in the OR. As this task is challenging due to the rotations and translations of the lamp camera relative to the patient caused by the surgeons and the dark lighting conditions during laparoscopic interventions, we use a second camera to capture the external surgical scene from a different point of view.

### Data set

**Fig. 2:**
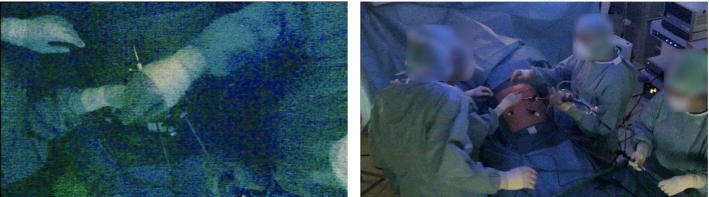
Example of time-synchronized frames of each camera. The image of *C*1 is shown on the left, and the one of *C*2 is on the right. Note that the frame of *C*1 has undergone image enhancement

We created a data set for the training of the extra-abdominal trocar and instrument detection. For this, we recorded four cholecystectomies with two time-synchronized extra-abdominal 2D cameras at the University Hospital rechts der Isar. The first camera (*C*1) recorded close-up images of the surgical area, while the second camera (*C*2) recorded the external surgical scene including the surgeons from a distant point of view. Figure 2 shows an example image of each camera view. Both cameras provided a resolution of $1920\times 1080$ pixels at 30 frames-per-second (fps). The videos of *C*1 were labeled at 6 fps together with medical experts for trocar object detection and binary annotated with information about the occupancy state of each trocar. Due to variable lighting conditions in laparoscopic interventions and the poor visibility of many trocars under dark ambient conditions, we uniformly increased the brightness of the frames of *C*1 for image preprocessing. This facilitated the annotation of these frames.

### Network architecture

**Fig. 3:**
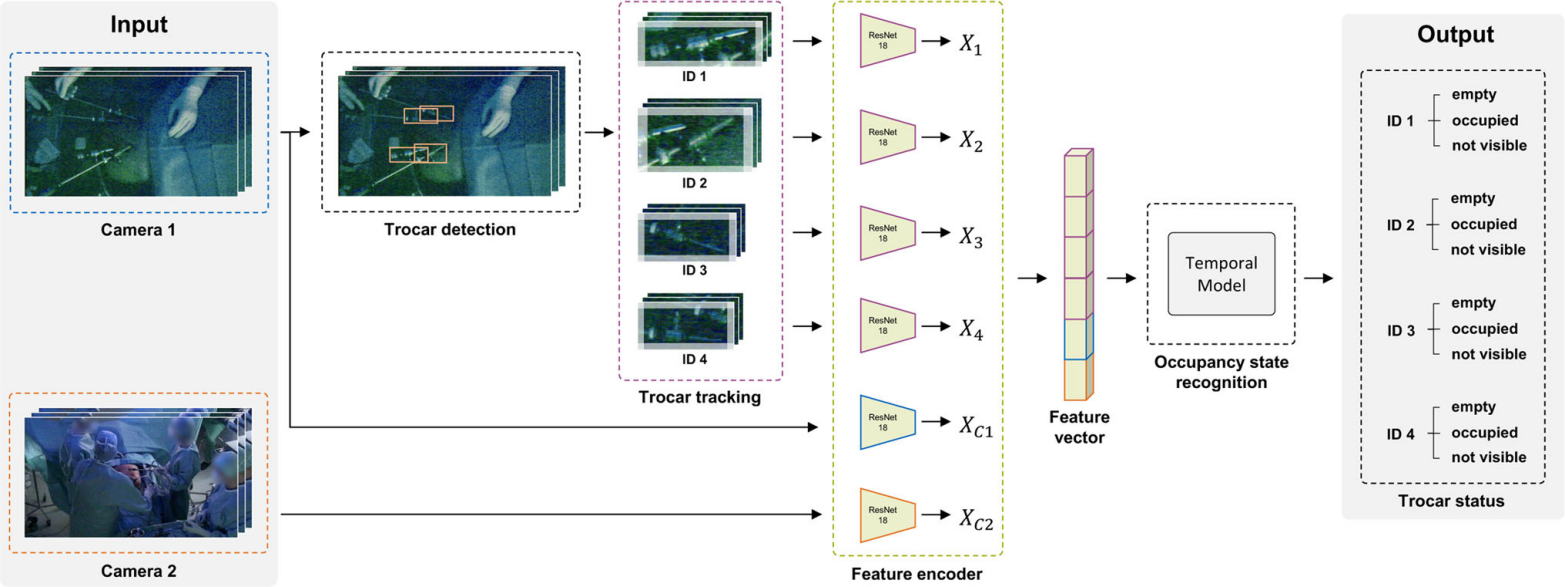
Overview of the network architecture. Trocars are detected using YOLOv8 based on images from camera 1. A centroid tracker assigns unique identifiers to each trocar, which are then subject to adaptive padding and encoded using ResNet18. The features of the cropped trocars ($X_1$, $X_2$, $X_3$ and $X_4$) are concatenated with the encoded features of camera 1 ($X_{C1}$) and camera 2 ($X_{C2}$). This combined feature vector is fed into the temporal model, which provides the occupancy state of each trocar

Our proposed model consists of three stages. A YOLOv8 [13] model detects the individual trocars on the images provided by camera 1. Using a centroid tracker, we assign an ID to each trocar and keep track of their positions as they move through successive frames of the videos. The detected trocars are cropped and subsequently encoded with adaptive padding using ResNet18 [14]. The features of the cropped trocars ($X_1$, $X_2$, $X_3$ and $X_4$) are concatenated with the encoded features of camera 1 ($X_{C1}$) and camera 2 ($X_{C2}$) in a joint feature vector. A temporal model classifies the occupancy state (empty, occupied or not visible) of the four trocars for each time *t* on basis of the feature vector. The full network architecture is illustrated in Fig. 3. Its image processing components are briefly presented below.

#### Trocar detection

For detection of the trocars and their 2D coordinates in terms of bounding boxes, we use a YOLOv8 model. By means of the 2D coordinates and the bounding box size of each trocar, the images are cropped with adaptive padding to the area of interest.

#### Trocar tracking

Based on the 2D coordinates, we compute the centroid for each detected trocar. A centroid tracker is employed to compare the centroids of the trocars in the current image with those of the previous images. Thereby, an ID is assigned to each trocar based on the Euclidean distance. When assigning identifiers, the tracker also takes bounding box areas into account, making it more stable against camera shifts. We use the prior knowledge that the number of trocars to be tracked during the entire period is limited to a maximum of four. By establishing an identifier assignment constraint, we simplify the object tracking process and increase the accuracy of the tracking algorithm.

#### Occupancy state recognition

The temporal model handles sequences of trocar crops and sequences of full images from *C*1 and *C*2 by initially encoding each image separately via a ResNet18. The resulting feature vectors are concatenated and fed into the temporal classifier for further processing. The network captures temporal relationships within the concatenated feature vectors to provide a contextual understanding of the input sequences. A classification head takes the network outputs and predicts the occupancy state for each trocar.We compare different models for temporal refinement such as an LSTM [15], gated recurrent unit (GRU) [16] and a multistage temporal convolutional network (MS-TCN) [17].

### Experimental setup

#### Model training

We trained the YOLOv8 model for trocar detection using the standardized YOLOv8 loss function for 30 epochs. The stochastic gradient descent optimizer, a learning rate of 1e-3 and a batch size of 16 were utilized. During training, the images were resized to $640 \times 640$ pixels and augmentations such as color space adjustment, affine transformations and horizontal and vertical flip were enabled.

The temporal models (LSTM, GRU and MS-TCN) were trained to recognize the occupancy state of the trocars, whether a laparoscopic instrument is inserted or not. The models were trained with a batch size of 32, a hidden layer size of 128 and a sequence length of 12. We used the multiclass focal loss [18] to handle the imbalance in the class distribution of empty (10.72%), occupied (65.35%) and not visible (23.93%) trocars. All models were implemented in PyTorch and trained on an NVIDIA RTX A6000.

#### Evaluation metrics

To assess the performance of our network architecture, we report common classification metrics such as the precision, recall and F1 score for both the trocar detection and the recognition of the occupancy state. By providing the metrics of the YOLOv8 network, an assessment of the performance of the temporal models can already be made, since these networks rely on the trocar detections to construct the feature vector.

## Results

### Trocar detection

We report the average evaluation results of the trocar detection algorithm’s performance over 4 folds on the extra-abdominal surgical videos of camera $C_1$. The YOLOv8 model achieved a precision of $95.18\pm 1.39\%$, indicating that $95.18\%$ of the instruments detected were true positives. The recall rate was $94.98\pm 1.30\%$, reflecting the algorithm’s ability to identify $94.98\%$ of all actual trocars present in the test data set. The F1 score as the harmonic mean of precision and recall amounted to $95.06\pm 0.88\%$.

**Table 1:**
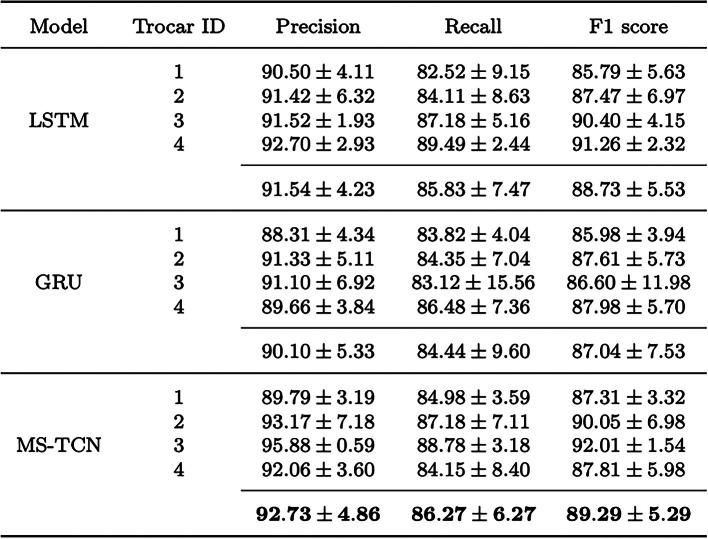
Comparison of different temporal models for occupancy state recognition of each trocar. We report the average metrics (%) using a 4-fold cross-validation along with their respective standard deviation (±) for each trocar. Mean values of each temporal model over all trocars are also provided

Bold values represent the best mean values

**Table 2:**
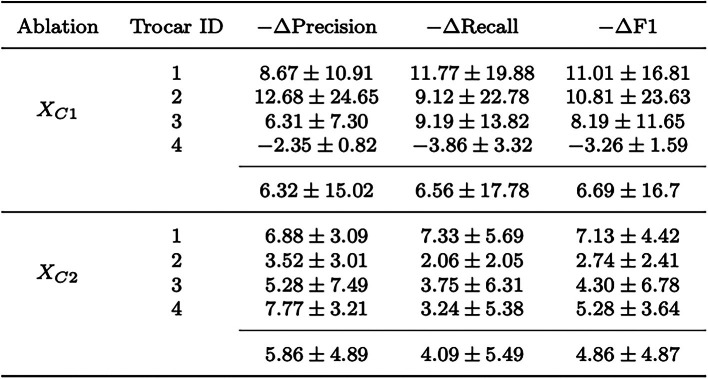
Changes in precision, recall and F1 score for occupancy state recognition as a result of ablative training of the temporal model MS-TCN. The changes of the metrics over 4 folds are reported (%) with the corresponding standard deviation (±)

### Occupancy state recognition

The results of the occupancy state recognition are shown in Table 1. We report the average metrics (%) using a 4-fold cross-validation along with their respective standard deviation (±) for each trocar and temporal model. In addition, we report the average of precision, recall and F1 score over all trocars for each temporal model, indicating the overall performance of the respective temporal models. The best performance for occupancy state recognition is achieved by the MS-TCN. The LSTM and GRU architectures reach a lower performance. The MS-TCN obtains a precision of $92.73 \pm 4.86\%$, showing that $92.73\%$ of the states (empty, occupied or not visible) were correctly classified, and achieves a recall rate of $86.27\pm 6.27\%$. The F1 score consequently amounts to $89.29\pm 5.29\%$. The GRU model exhibits the worst values and the highest standard deviation for the average precision, recall and F1 score in this comparison.

Considering the occupancy state recognition for each trocar individually, the MS-TCN performs best in most cases. The LSTM reaches better metric scores for trocar 4 and better precision for trocar 1. These trocars correspond to the two working trocars of the surgeon, which are exposed to more movements. In all cases, the precision is slightly higher than the recall.

### Ablation study

To assess the effect of using the full camera image of *C*1 and *C*2 as an input of the temporal model, we trained the model with the best performance in our comparison, MS-TCN, excluding the full images captured by *C*1 and *C*2, respectively. Therefore, the training for the MS-TCN was repeated without $X_{C1}$ or $X_{C2}$ in the concatenated feature vector. In Table 2, we report the magnitude changes in the model’s output regarding precision, recall and F1 score for each trocar. Furthermore, the average of precision, recall and F1 score over all trocars for both ablation trainings are given.

Excluding $X_{C1}$ or $X_{C2}$ results in a drop in the metric scores, where the overall magnitude changes are higher for *C*1 than for *C*2. Only for trocar 4 an ablation of $X_{C1}$ leads to an improvement of the metric values. In general, the removal of $X_{C1}$ has a more pronounced impact on the performance of the model, especially for trocar 1 and 2.

## Discussion

Our work establishes a first step towards extra-abdominal situational awareness in laparoscopic settings by focusing on the identification and occupancy state detection of trocars during laparoscopic procedures. We propose a three-stage architecture consisting of a YOLOv8 network to detect the trocars, a centroid tracker to assign a unique identifier and functionality to the trocars and a temporal model recognizing the occupancy state of each individual trocar.

The YOLOv8 model achieves remarkable results considering the difficult lighting conditions in the operating room. Despite the required brightening of C1’s images to make labeling possible, the detection algorithm achieves an F1 score of $95.06\pm 0.88\%$ over 4 folds. This high value provides a solid foundation for the occupancy state recognition, as the feature vector for the temporal model relies on the trocar detection.

The comparison of the three different temporal models for the occupancy state recognition revealed that the best result could be obtained by the MS-TCN architecture, reaching an F1 score of $89.29\, \pm \, 5.29\%$. The score is slightly lower than for the trocar detection, as the recognition relies on the upstream detection. The small gap indicates that the temporal model is performing effectively.

The ablation study shows that removing $X_{C1}$ from the feature vector causes the largest drop of the metric values for trocars 1 to 3, while there is an improvement for trocar 4. The high variance indicates that the model is very sensitive to variations in the training data set, which can be attributed to its small size. Ablation of $X_{C2}$ leads to a decrease in metric scores for all trocars, with the surgeon’s working trocars (1 and 4) showing a higher drop. Overall, this shows that using the full camera images improves the model’s output.

However, there are still a number of challenges to overcome before real-time embedding of the algorithm in the operating room is possible. Increasing the size of the data set is an essential step towards enhancing the algorithm’s reliability, as only four laparoscopic surgeries were considered in this article. This will make the algorithm more robust to camera movements, such as rotation and translation relative to the patient, and ensure accurate tracking and assignment of trocar functionalities.

In the future, consideration should therefore be given to a camera replacement, mitigating movement and occlusion. In addition, improved filter properties of the camera, such as installed neutral-density filter, should ensure better data quality when recording the data set. To achieve a substantial improvement of context awareness for assistive systems in the operating room, we would like to incorporate the laparoscopic video as a next step. This will allow us not only to identify the presence of instruments within the abdomen, but also to identify which specific instrument is inserted in each trocar.

## Conclusion

In this article, we present a deep learning-based three-stage architecture that detects and tracks trocars and outputs the trocar occupancy state based on extra-abdominal cameras. The proposed model was trained on four recorded cholecystectomies. We achieve an F1 score of $95.06\pm 0.88\%$ for the trocar detection with YOLOv8 and an F1 score of $89.29\pm 5.29\%$ for the prediction of the occupancy state with an MS-TCN. Moving forward, we intend to improve the results by creating a larger data set with an optimized camera setup and integrating time-synchronized laparoscopic videos to be able to assign specific instrument information to each occupied trocar. Furthermore, we envision developing strategies for real-time deployment in the OR to enhance the surgical workflow understanding.

## Data Availability

The data that support the findings of this study are available on request from the corresponding author. The data set is not publicly available due to restrictions related to privacy concerns for the research participants.
